# First Report of Benzimidazole Resistance in Field Population of *Haemonchus contortus* from Sheep, Goats and Cattle in Bosnia and Herzegovina

**DOI:** 10.3390/pathogens13010077

**Published:** 2024-01-15

**Authors:** Naida Kapo, Jasmin Omeragić, Šejla Goletić, Emina Šabić, Adis Softić, Ahmed Smajlović, Indira Mujezinović, Vedad Škapur, Teufik Goletić

**Affiliations:** 1 Veterinary Faculty, University of Sarajevo, 71000 Sarajevo, Bosnia and Herzegovina; jasmin.omeragic@vfs.unsa.ba (J.O.); sejla.goletic@vfs.unsa.ba (Š.G.); eminasabic123@hotmail.com (E.Š.); adis.softic@vfs.unsa.ba (A.S.); ahmed.smajlovic@vfs.unsa.ba (A.S.); indira.mujezinovic@vfs.unsa.ba (I.M.); teufik.goletic@vfs.unsa.ba (T.G.); 2 Faculty of Agriculture and Food Science, University of Sarajevo, 71000 Sarajevo, Bosnia and Herzegovina; v.skapur@ppf.unsa.ba

**Keywords:** benzimidazole resistance, *Haemonchus contortus*, sheep, goats, cattle, rt-qPCR

## Abstract

*Haemonchus contortus* is a globally significant parasitic nematode in ruminants, with widespread resistance to benzimidazole due to its excessive and prolonged use. Given the extensive use of benzimidazole anthelmintics in Bosnia and Herzegovina, we hypothesized that resistance is prevalent. The aim of this study was to identify the presence of anthelmintic resistance to benzimidazole in *H. contortus* from naturally infected sheep, goats and cattle in Bosnia and Herzegovina through the detection of the Phe/Tyr polymorphism in the amino acid at position 200 of the β-tubulin protein. From 19 locations in Bosnia and Herzegovina, a total of 83 adult *H. contortus* were collected from the abomasum of ruminants. Among these, 45 *H. contortus* specimens were isolated from sheep, 19 from goats and 19 from cattle. Results showed that 77.8% of *H. contortus* in sheep exhibited homozygous resistant genotypes at position 200 of the β-tubulin gene, with 15.5% being heterozygous. In goats, all tested *H. contortus* (100%) were homozygous resistant, and no heterozygous resistant or homozygous sensitive genotypes were found. Cattle had 94.7% homozygous resistant *H. contortus*, with no heterozygous resistant genotypes detected. In *H. contortus* from sheep and cattle, 6.7% and 5.3%, respectively, displayed homozygous sensitive genotypes. This study, for the first time, highlights the presence of a resistant population of *H. contortus* in sheep, goats and cattle in Bosnia and Herzegovina, using the rt-qPCR method. The resistance likely spread from sheep or goats to cattle, facilitated by shared pastures and the practice of transhumance, indicating a widespread and growing issue of anthelmintic resistance.

## 1. Introduction

Parasitic infections caused by gastrointestinal nematodes (GIN) are one of the leading causes of economic losses in livestock production worldwide [1]. Within the *Trichostrongylidae* family, parasites of the *Haemonchus* genus are considered the most pathogenic and economically significant among ruminant parasites, primarily due to their intense blood feeding and parasitic habits. 

The *Haemonchus* genus includes more than 10 species, with *Haemonchus contortus* and *Haemonchus placei* standing out as the two most commonly encountered helminths. *H. contortus* primarily infects grazing sheep and goats, while *H. placei* is predominantly found in larger ruminants such as cattle. However, both species have been identified in various domesticated or wild ruminant populations and have been observed in infections, either mixed or as single species, demonstrating their adaptability and broad host range [2,3,4].

The life cycle of *H. contortus* which unfolds over a three-week period, includes both parasitic and free-living phases. After the ingestion of infective third-stage larvae (L3s), the impacts of fourth-stage larvae and adult *H. contortus* become particularly pronounced for hosts. This influence is mainly attributed to their blood feeding activity in the abomasum, resulting in anemia, complications and often the demise of heavily infected animals [5]. This parasitic cycle has profound implications for animal productivity, characterized by inadequate weight gain, weight loss and diminished wool and milk production, as well as weakness or compromised health [6,7]. The consequences can be severe, with potential sudden fatalities, given its highly pathogenic nature as a blood-feeding species [8].

Recommendations for best practice control of GIN have undergone substantial transformations on a global scale over the past decades. With the advent of well-toleratedbroad-spectrum anthelmintics, control strategies have shifted towards frequent administration of chemical anthelmintics to the entire flock [9]. Among the key anthelmintics used to combat infections caused by these and other GIN species in both large and small ruminants are benzimidazoles (BZ) [10,11]. However, the frequent and indiscriminate use of BZ anthelmintics has led to diminished efficacy and the development of resistance [12]. Therefore, the effectiveness of anthelmintics is increasingly jeopardized by the widespread emergence of resistance to these anthelmintics in *H. contortus* populations worldwide [13]. Furthermore, the rise of anthelmintic resistance (AR) is a prevalent issue in Europe, with documented cases of BZ-resistant *H. contortus* in various countries, including Sweden [14,15], the Netherlands [16], Belgium [17], Germany and Switzerland [18], among others. This issue is particularly significant in regions where intensive and extensive livestock farming is crucial for sustaining rural agriculture [19], and where new classes of anthelmintics have thus far been inaccessible or prohibitively expensive, as is the case in Bosnia and Herzegovina (B&H). 

Detection and monitoring of AR in ruminants often rely on various methods, with in vivo tests like FECRT considered the standard but time-consuming and expensive to use on a routine basis [20]. Similarly, in vitro tests, while developed, are characterized by relatively low sensitivity [21]. Today, molecular assessment of BZ resistance is considered the most sensitive and advanced approach for detection [22]. Specifically, nematode resistance to BZ can be identified through molecular tests that detect single nucleotide polymorphisms (SNPs) in the gene encoding β-tubulin isotype-1, located at codons 200, 167 and 198. In the case of *H. contortus*, SNPs at codon 200 (i.e., TTC to TAC) [23] and 167 (i.e., TTC to TAC) [24] lead to the substitution of phenylalanine with tyrosine, while codon 198 (i.e., GAA to GCA) results in the substitution of glutamic acid with alanine [25,26,27]. All three SNPs are considered to be associated with BZ resistance. Among them, SNP 198 is considered the least common of the three mutations associated with BZ resistance. Fewer studies have confirmed the presence of this mutation compared to the mutations at positions 200 and 167. The rt-qPCR method further accelerates the detection of BZ resistance in various GIN species. This allows for a swift response to alter treatment methods and enhance deworming strategies.

In B&H, research on AR in nematodes of both large and small ruminants has never been conducted. The issue of AR is particularly significant for sustainable sheep and goat farming. Various data indicate a growing incidence of resistance in large ruminants, which can potentially become a major concern for cattle production. Furthermore, the practice of transhumance, the seasonal movement of livestock to different pastures within the country, still exists in B&H [28]. This practice contributes to the spread of resistant parasites between small and large ruminants and the broader establishment of resistant nematode populations. 

The primary objective of this study was to assess the prevalence of BZ resistance in *H. contortus*, derived from sheep, goats and cattle across 19 locations in B&H. The investigation focused on identifying the Phe/Tyr polymorphism within the amino acid sequence at position 200 of the β-tubulin protein. This research aimed to provide insights into the potential development and spread of BZ resistance among these ruminant species, contributing valuable information for the management and control of parasitic infections in livestock.

## 2. Materials and Methods

### 2.1. Study Area

**Figure 1:**
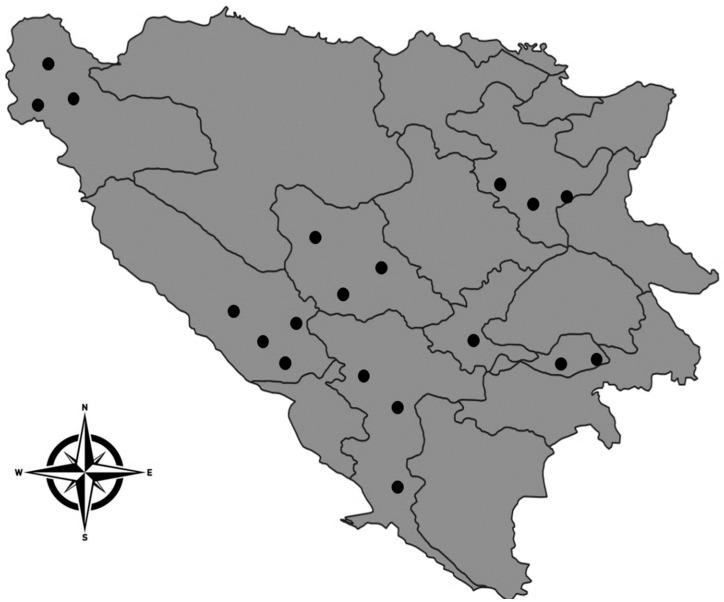
Sampling locations across B&H, from which abomasum samples were collected from sheep, goats and cattle.

In this study conducted during the 2021/2022 season, a total of 407 abomasum samples were collected. This included 200 samples from sheep, 47 from goats and 160 from cattle, originating from 19 locations in B&H that are notable for extensive livestock farming (Figure 1). Abomasum sampling was carried out during the regular slaughter of adult sheep, goats and cattle on farms under veterinary supervision and no experimentation on animals has been conducted to obtain data presented in this paper. Carcasses for material collection were selected randomly. The abomasum of each animal was removed and dissected. After sampling, following the cold chain transport protocols, the samples were sent to the laboratories of the Veterinary Faculty in Sarajevo.

### 2.2. Helminths

The contents of the abomasum (a total of 407) were removed by gently scraping the abomasal mucosa to obtain the ingested food content and any potential parasites retained on the abomasal mucosa. The collected abomasum contents were filtered through a mesh or sieve to separate solid waste and larger particles from the liquid part. The liquid containing larger particulates was allowed to settle to ensure that the parasites settled at the bottom. A portion of the sediment at the bottom of the container was examined section by section under the microscopes OLYMPUS (CH20BIMF200, Tokyo, Japan) and Leica (model EZ4, Wetzlar, Germany) [29,30]. 

### 2.3. Identification of H. contortus

Samples of *Haemonchus* were obtained from animals confirmed to be infected with *Haemonchus* parasites. Adult *H. contortus* were identified based on their morphological characteristics as described by Soulsby [31] and Lichtenfels et al. [32]. Randomly selected *H. contortus* were chosen for further analysis (45 from sheep, 19 from goats and 19 from cattle). Following the identification of *H. contortus* parasites, material preparation for molecular diagnostics was conducted.

### 2.4. Parasite Populations and Genomic DNA Isolation

Eighty-three adult *H. contortus* specimens were removed from ethanol, air-dried and stored at −20 °C in 1.5 mL tubes without a buffer. The head of each helminth was dissected at the cervical papillae for analysis, excluding the parasite’s uterus and eggs, and collected for DNA extraction. Samples were homogenized for 2 min at 25 Hz using a TissueLyser II (Qiagen, Venlo, The Netherlands), followed by centrifugation at 14,000× *g* for 2 min. DNA extraction was performed with the DNeasy Blood & Tissue Kit^®^ (Qiagen, Hilden, Germany) according to the manufacturer’s instructions. The extracted DNA was eluted in 50 μL of nuclease-free water and stored at −20 °C until further use.

### 2.5. The Real-Time Quantitative PCR for Confirming H. contortus

The real-time quantitative PCR (rt-qPCR) assay for the detection of *H. contortus*, as described by Von Samson-Himmelstjerna et al. [33], was performed using genus-specific primer and species-specific probe combinations derived from the second internal transcribed spacer (ITS2) ribosomal DNA transcription of *H. contortus* (Table 1). The master mix consisted of 10 µL of 2x QuantiTect Probe RT-PCR Master Mix (Qiagen, Hilden, Germany), 3.5 µL of RNase-free water, 5 µL of the sample and 1.5 µL of *H. contortus*-specific primer-probe mix. The final concentration of the primers in the *H. contortus*-specific primer-probe mix was 0.7 µM, and the final concentration of the probe was 0.2 µM. RNAse-free water was used as a negative control. The cycling protocol consisted of the initial denaturation at 95 °C for 3 min, followed by 40 cycles of denaturation at 95 °C for 50 s, annealing at 57 °C for seconds, and extension at 72 °C for 50 s. The fluorescence was measured at the end of each annealing step. The qPCR platform used was the Magnetic Induction Cycler (MIC qPCR) from Bio Molecular Systems and the results were analyzed using the MicPCR Software v2.2.0.

### 2.6. The Real-Time Quantitative PCR for Resistance Detection

The rt-qPCR method was employed to determine homozygous and/or heterozygous resistant genotypes based on the presence of the amino acid residue Phe at position 200 of the β-tubulin gene in *H. contortus*, using primers previously published by Humbert and Elard [34] and Arsenopoulos et al. [22].

For amplification of allele-specific products, four primers were used as follows: two allele-nonspecific primers (P1 and P4) for species detection and two allele-specific primers for BZ resistance in *H. contortus* (P2S and P3R) (Table 2) (Eurofins Genomics Germany GmbH, Ebersberg, Germany). This approach generated three fragments, each varying in length: P1/P4 primer combination generates a fragment specific to the species (827 bp), P3R/P4 primer combination generates a fragment specific to a resistant allele (635 bp) and P1/P2S primer combination generates a fragment specific to a susceptible allele (242 bp). All four primers were pooled together in a single primer mix, in which the concentration of P1 and P4 primers was 8 µM, and the concentration of P2S and P3R was 10 µM.

The master mix consisted of 10 µL 2X Fast EvaGreen Master Mix (Biotium, Fremont, USA), 4 µL RNase free water, 5 µL of the sample and 1 µL of a specific primer mix in which the final concentrations of the P1 and P4 primers were 0.4 µM, and the final concentrations of the primers PS2 and P3R primers were 0.5 µM. RNase-free water was used as a negative control. The cycling protocol included an initial step of denaturation at 95 °C for 2 min, followed by 45 cycles consisting of denaturation at 94 °C for 30 s, annealing at 57 °C for 30 s, extension at 72 °C for 45 s and final extension at 72 °C for 10 min. This was immediately followed by the melting curve analysis, during which the temperature was gradually increased from 65 °C to 95 °C, with an increment of 0.3 °C per cycle. Fluorescence signals were measured and recorded at the end of each extension step, as well as continuously during the melting point analysis. The qPCR platform and the software for analysis used were the same as for rt-qPCR detection of *H. contortus.*

### 2.7. Data Management and Analysis

The frequency of susceptible/susceptible, resistant/susceptible and resistant/resistant helminths recovered from the animals was compared. The statistical analysis of the molecular diagnostics results was performed using Minitab 17 software [35]. Differences in the frequency of positive findings between animal species or between locations were analyzed using the χ² test. Statistical significance was defined at *p* < 0.05.

## 3. Results

**Figure 2:**
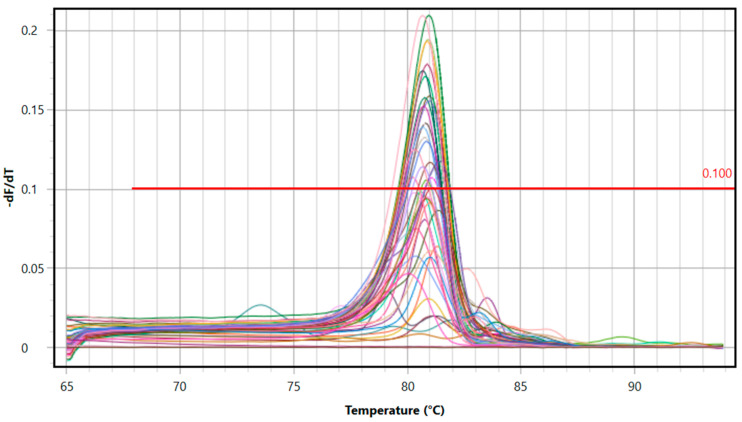
The Tm (melting temperature) values of the amplicons were obtained by analyzing their dissociation characteristics, with continuous heating and fluorescence measurement in a temperature range of 65 °C to 95 °C, following the completion of the EvaGreen^®^ rt-qPCR method. Clear peaks of the tested sample amplicons and significantly different Tm values of the samples (Tm = 83.2–84.3 °C) compared to the PCR water used as a negative control (70.2 °C) confirm the specificity of the amplification process.

The rt-qPCR results confirmed that all samples were *H. contortus*. Out of the 83 *H. contortus* helminths evaluated during this study, 72 (86.8%) were found to be homozygous resistant, 7 (8.4%) were found to be heterozygous resistant and 4 (4.8%) were found to be homozygous susceptible (as determined by targeting position 200 of the β-tubulin protein of the helminths) (Table 3). The obtained melting temperature (Tm) and dissociation curves are presented in Figure 2.

There was no statistically significant difference in the frequency of SS, RS and RR genotypes among the three animal species (sheep, goats and cattle) from which the parasites were collected (*p* = 0.08815). Therefore, in this case, the animal species had no influence on SS, RS and RR. Statistical analysis of the determined values between small (sheep and goats) and large (cattle) ruminants revealed no difference in the frequency of the SS, RS and RR genotypes of *H. contortus* found in sheep and goats (*p* = 0.081916), sheep and cattle (*p* = 0.17714) and goats and cattle (*p* = 0.598389). Thus, it was further confirmed that the animal species had no influence on SS, RS and RR (Table 3).

## 4. Discussion

Benzimidazoles, widely used globally as anthelmintics against parasites in domestic ruminants and humans [36], exhibit a broad spectrum of anthelmintic activity, cost-effectiveness and a short withdrawal period [37]. However, their extensive use has led to the development of resistance and reduced efficacy against GIN, including *H. contortus* [38]. Despite the crucial roles of sheep, goats and cattle in B&H, the issue of BZ resistance in *H. contortus* helminths from these animals had not been explored until this study. Using rt-qPCR, we investigated the presence of the F200Y mutation associated with BZ resistance in *H. contortus*. This research marks the first identification of the F200Y mutation in *H. contortus* in sheep, goats and cattle in B&H, employing rt-qPCR and other molecular methods for phenotypic confirmation of BZ resistance. 

Overall, our results suggest that the F200Y mutation has been established in flocks of sheep, goats and cattle in the area of B&H. Similar findings have been confirmed in this part of southern Europe, notably in Greece [22,39]. Gallidis et al. [39] identified F200Y mutation in only a few GIN originating from sheep. In the latter study by Arsenopoulos et al. [22], the presence of homozygous and heterozygous resistant alleles was detected in 96.9% of *H. contortus*, while homozygous sensitive alleles at codon 200 were not confirmed. The results obtained in our research are similar to those of Arsenopoulos et al. [22]. In our study, 100% of *H. contortus* isolated from goats had homozygous resistant alleles, while in sheep it was 77.4%, and in cattle it was 94.7%. In the study by Arsenopoulos et al. [22], they confirmed the presence of 75.0% heterozygous resistant alleles and 25.0% homozygous resistant alleles in cattle. Furthermore, in sheep and cattle in the B&H, the presence of 6.7% and 5.3% homozygous sensitive alleles, respectively, was confirmed, in contrast to the study by Arsenopoulos et al. [22], where the presence of homozygous sensitive alleles in *H. contortus* was not detected.

In other studies, differences in the prevalence of resistant alleles have been observed among different countries and genera of parasites. The SNP 200 has been widely established in *H. contortus* isolates from the European continent, especially in countries such as Switzerland, Italy [40], Belgium [17], Hungary [41], the UK [42], Sweden [14,43], France [25] and Austria [44], as well as in parts of North America [45] and Asia [46]. In Switzerland, a significant increase in the frequency of resistance associated with the TAC mutation at codon 200 was observed on all tested sheep farms (31/31), while in Italy, resistance was confirmed to a lesser extent, on two out of seven farms (28.6%) [41].

In a study on *H. contortus* resistance in Hungary, the presence of 63.7% of homozygous resistant alleles at codon 200 (TTC/TAC) was confirmed [41]. In the regions of Styria and Salzburg in Austria, larvae of *H. contortus* from 16 sheep farms were examined, and pyrosequencing determined the presence of SNPs at codons 200, 167 (TTC/TAC) and 198 (GAA/GCA) of the isotype-1 β-tubulin gene [44]. The frequency of the F200Y substitution associated with resistance was confirmed in 87.0–100.0% of cases [44]. In Sweden, samples of *H. contortus* from 67 sheep farms were collected and analyzed and the presence of SNP 200 (TTC/TAC) was observed in a range of 14.1% to 100.0% [43]. Across the UK, *H. contortus* in sheep exhibited the presence of SNP 200 (TTC/TAC) on all seven tested farms, with it being the most common SNP, accounting for a total of 36.3%, except on one farm in North Yorkshire [42]. In Canada, SNPs associated with resistance to BZ were analyzed in *H. contortus* from fecal samples collected from 16 sheep farms in Ontario, Canada. Homozygous resistance at position 200 (TTC/TAC) was observed on 36.4–97.7% of the examined farms, while homozygous resistance at position 167 (TTC/TAC) was confirmed in a smaller percentage, 0.3–4.7%. Heterozygotes at both positions 200 and 167 (TTC/TAC) were observed on 1.4–9.2% of farms [45]. In order to gain deeper understanding of the nature and specifics of the established BZ resistance in B&H, it would be beneficial to conduct a comprehensive study aimed at investigation of the SNPs 167 and 198 of the β-tubulin gene as well as the phenotypic resistance of *H. contortus*.

While numerous studies have explored BZ resistance in *H. contortus* in sheep and goats, there are limited data on resistance in *H. contortus* isolated from cattle and other large ruminants. Particularly concerning is the confirmation of homozygous resistant genotypes of *H. contortus* (94.7%) in cattle in B&H. In an unpublished field survey, veterinarians and farmers in B&H identified BZ as the primary class of anthelmintics used for deworming in sheep, goats and cattle. It is noteworthy that sheep, goats and cattle graze on common pastures in B&H, exposing them to the same GIN. Federal Ministry of Agriculture, Water Management, and Forestry data [28] indicate prevalent and extensive livestock farming and transhumance in hilly and mountainous areas of B&H, likely contributing to high resistance across all three animal species. This is supported by the widespread presence of homozygous resistant genotypes observed in all examined areas. Our field study (unpublished data) also revealed that 63.8% of farmers reported practicing the translocation of animals to other pastures, potentially facilitating the spread of BZ resistant *H. contortus* among sheep, goats, and cattle and contributing to the broader dissemination of resistant helminths. Furthermore, it can be postulated that the confirmation of resistant *H. contortus* in cattle is a result of communal grazing rather than the direct action of anthelmintics [46,47]. 

There are only a few studies on the impact of transhumance on GIN infections in sheep, goats and cattle and its influence on the development of AR. Lamberzt et al. [48] confirmed that noteworthy indications suggest that shared characteristics of hill and mountain farming environments may foster the emergence of AR. Given that GIN infections significantly impact animal health and farm profitability [49], the widespread occurrence of AR could pose a severe threat to husbandry practices in B&H. In Austria, a comprehensive investigation was undertaken to assess the influence of transhumance on the spread of resistant parasites. The findings of the study point to a notable percentage of BZ-resistant *H. contortus* and *T. colubriformis* in Austrian sheep, aligning with reported inefficacy of BZ treatments in the region [44]. The recurring theme of “dose and move” practices in transhumance emerges as a potential influencer on the observed high frequency of resistance alleles in *H. contortus* in Austria [44]. This practice, common in transhumance, mirrors trends observed in other regions employing similar husbandry methods.

In light of the present results coupled with the notable fertility of *Haemonchus* [50], there is a considerable apprehension that the population of BZ-resistant *H. contortus* in B&H may continue to grow both in size and geographic extent. This expansion could be attributed to factors such as treatment errors, animal migrations and/or transmission through hosts from the wild [51].

In conclusion, we emphasize the importance of developing parasite control guidelines and management practices in the region and in Europe to reduce selection pressures for AR. It will also be crucial to develop parasite management strategies tailored to individual farms, including quarantine treatments for imported livestock, the use of appropriate drug combinations for strategic treatment, identifying parasite refugia in hosts with susceptible parasites and closely monitoring AR using molecular methods such as rt-qPCR. In the future, it would be beneficial to conduct a comprehensive in vitro study aimed at also investigating the phenotypic resistance of *H. contortus* as well as the other SNPs of the β-tubulin gene (positions 198 and 167) that are also conferring resistance to BZ these parasites. Moreover, in our opinion, comprehensive research on other resistances in parasitic nematodes in livestock is urgently needed to inform future integrated parasite control programs.

## Figures and Tables

**Table 1:** Primer and probe sequences for the detection of *H. contortus* [33].

Primer-probe	Sequence
Hc 2 multi 307T	5′-FAM-TGGCGACGATGTTC-MGB-3′
Hc 2 multi 272F	5′-GCGAATATTGAGATTGACTTAGATAGAGAC-3′
Hc 2 multi 349R	5′-GCTCAGGTTGCATTATACAAATGATAAA-3′

**Table 2:** Primers used for rt-qPCR for targeting position 200 of the β-tubulin protein of the *H. contortus* helminths [22,34].

Primer	Sequence	Source
P1	Fw: 5′-GTCCCACGTGCTGTTCTTGT-3′	[22]
P2S	Rv: 5′-TACAGAGCTTCATTATCGATGCAGA-3′	[22]
P3R	Fw: 5′-TTGGTAGAAAACACCGATGAAACATA-3′	[22]
P4	Rv: 5′-GATCAGCATTCAGCTGTCCA-3′	[22]

**Table 3:** Presentation of determined homozygous and heterozygous resistant (RR and RS) and susceptible (SS) genotypes of *H. contortus* isolated from sheep, goats and cattle in B&H.

Species	SS	RS	RR	Total
Sheep	3, (6.7%)	7, (15.5%)	35, (77.8%)	45
Goats	0, (0.0%)	0, (0.0%)	19, (100.0%)	19
Cattle	1, (5.3%)	0, (0.0%)	18, (94.7%)	19
Total	4, (4.8%)	7, (8.4%)	72, (86.8%)	83

The variations in SS, RS and RR genotypes of *H. contortus* across different locations did not show statistical significance for sheep (*p* = 0.3333), goats (*p* = 1) or cattle (*p* = 0.534742).
